# Pattern of recurrence after fractionated stereotactic reirradiation in adult glioblastoma

**DOI:** 10.1186/s13014-025-02611-0

**Published:** 2025-02-28

**Authors:** Agathe Margulies, Nassim Sahki, Fabien Rech, Guillaume Vogin, Marie Blonski, Didier Peiffert, Luc Taillandier, Grégory Lesanne, Nicolas Demogeot

**Affiliations:** 1 Department of Radiotherapy, Institut de Cancérologie de Lorraine, Vandoeuvre-lès-Nancy, 54519 France; 2 Faculté de Médecine de Nancy, Université de Lorraine, 9 avenue de la Forêt de Haye, Vandoeuvre-lès-Nancy, 54505 France; 3 Biostatistic Unit, Institut de Cancérologie de Lorraine, Vandoeuvre-lès-Nancy, 54519 France; 4 Department of Neurosurgery, CHRU-Nancy, Nancy, 54000 France; 5 Department of Radiotherapy, Centre François Baclesse, Esch-sur-Alzette, Luxembourg; 6 Department of Neurology, CHRU-Nancy, Nancy, 54000 France; 7 Department of Radiology, Institut de Cancérologie de Lorraine, Vandoeuvre-lès-Nancy, 54519 France; 8 Institut de Cancérologie de Lorraine – Alexis-Vautrin Cancer Center – Unicancer Academic Department of Radiation Therapy & Brachytherapy, 6 avenue de Bourgogne – CS 30 519, Vandoeuvre-lès-Nancy, cedex F-54 511 France

**Keywords:** Glioblastoma, Recurrence, Cyberknife, Stereotactic radiotherapy, Reirradiation, Pattern of recurrence

## Abstract

**Background:**

Glioblastomas all eventually relapse after initial treatment, and an option to treat these recurrences is fractionated stereotactic reirradiation (fSRT). The location of recurrences following reirradiation has not been studied for fSRT delivered by a dedicated stereotactic device. We aimed to analyze these locations to better elucidate safety margins, dose and fractionation regimens.

**Methods:**

We retrospectively analyzed the data of patients with glioblastoma recurrence that had been reirradiated by fSRT in October 2010-December 2020, in 25 Gy in 5 fractions delivered by a CyberKnife^®^ at Institut de Cancérologie de Lorraine. We matched the images of the post-fSRT relapse with the stereotactic radiation treatment planning scan to determine the relapse location.

**Results:**

The location of recurrences after fSRT was “out-field” in 43.5%, “marginal” in 40.3%, and “in-field” in 16.1% of patients (*N* = 62). A GTV-PTV margin of 1 mm (versus 2–3 mm, HR = 0.38 [0.15–0.95], *p* = 0.037) and a PTV volume of ≥ 36 cc (HR = 5.18 [1.06–25.3], *p* = 0.042) were significantly associated with the “marginal” recurrences. Being ≥ 60 years old at initial treatment (HR = 3.06 [1.17–8.01], *p* = 0.023) and having one or more previous recurrences (HR = 5.29 [1.70–16.5], *p* = 0.004) were significantly associated with “out-field” recurrences. The median PFS from fSRT was 3.4 months, and OS from diagnosis and from fSRT were 25.7 and 10.8 months respectively.

**Conclusion:**

Reirradiation of glioblastoma recurrence by fSRT with 25 Gy in 5 fractions provides good local control.

## Introduction

Glioblastomas are aggressive tumors, and they all eventually relapse after initial treatment. At present, there is no standard of care for these recurrences, but many options are available, alone or in combination: surgery, systemic therapies such as chemotherapy or antiangiogenic agents, tumor treating fields (TTF), and reirradiation, in conjunction with supportive care [1–10]. Among these options, reirradiation, including fractionated stereotactic reirradiation (fSRT) [11, 12], has been widely studied, and its safety and efficacy have been demonstrated [13–16].

Only a few studies have examined the location of glioblastoma recurrences after reirradiation – that is, tumor location following treatment for a previous relapse. Niyazi et al. observed glioblastoma location after conventional radiation therapy [17], and Shapiro et al. after fSRT delivered by a LINAC [18]. When fSRT is not delivered by a dedicated stereotactic device, the dose gradient is less important and may decrease local control. Also, both studies only considered patients who received bevacizumab concurrent to the reirradiation. In these studies, the recurrences were mainly located “in-field”, i.e., if more than 80% of the tumor recurrence was within the prescription isodose surface (95% in these cases) [17, 18]. Otherwise, they were distributed among two other categories: “marginal” (i.e., 20–80% of the tumor recurrence included in the prescription isodose surface) or “out-field” (i.e., less than 20% of the tumor recurrence included in the prescription isodose surface).

A better knowledge of the location of recurrences would allow an adaptation of treatment: increasing the radiation dose if recurrences are mostly located in-field, or increasing safety margins if they are mostly marginal.

In our study, we retrospectively analyzed the patterns of glioblastoma recurrence after fSRT, with or without a concurrent systemic agent, as there are limited data to inform the choice of safety margins as well as dose and fractionation regimen.

## Materials and methods

### Study design

Included patients had to meet the following inclusion criteria: adult over 18 years old; initial diagnosis of histologically proven glioblastoma (histological grade IV, OMS classification); initial treatment according to international recommendations by surgery (gross total resection, subtotal resection or biopsy) followed by radiotherapy (with conventional dose and fractionation regimen of 60 Gy in 30 fractions) and concomitant temozolomide, then adjuvant systemic treatment; first in-field recurrence treated by fSRT (Cyberknife^®^ (Accuray Inc.)); fSRT treatment occurred from October 2010 to December 2020 wether it was the first recurrence at all or not, at *Institut de Cancérologie de Lorraine*; and recurrence after reirradiation. The recruitment was retrospective, patients’ data were collected into hospital database and this study was approved by the local institutional research ethics committee and registered with the French Data Protection Authority (“Commission Nationale de l’Informatique et des Libertés”).

The recurrence was assessed with RANO (Response Assessment in Neuro-Oncology) criteria [19] on magnetic resonance imaging (MRI), but only T1-enhanced lesions were considered in our study.

The treatment was described in a previous study conducted in our hospital [20], with fSRT delivered by a dedicated stereotactic radiation therapy machine (Cyberknife^®^ (Accuray Inc.)), with 5 sessions of 5 Gy, distributed over 5–7 days, prescribed to the 80% normalized isodose line. The gross tumor volume (GTV) corresponded to the T1-enhanced lesion(s) on a recent brain MRI (maximum 4 weeks before treatment), matched with simulation computed tomography (CT). The planning target volume (PTV) was defined by an isotropic margin of 1–3 mm added to the GTV. Organs at risk included optic nerve, optic chiasma, brainstem, lens, pituitary, and healthy brain. Concurrent or adjuvant systemic therapy was prescribed in some cases according to regional neurooncology tumor board decision.

The clinical follow-up included an MRI at 1 and 3 months after reirradiation and at least every 3 months afterwards.

The analysis was performed on all patients, regardless of the delay between reirradiation and progression, but also on a subgroup of patients who had recurrences at least 90 days after reirradiation, to avoid a confounding bias with pseudoprogression.

### Data collection

The different treatments characteristics, the demographics, and clinical data at every stage of treatment were retrieved from medical records. Subventricular zone invasion was determined on the diagnostic MRI. Intracranial hypertension, sensorimotor defect, headache, use of steroids and dosage were recorded at recurrence. The Karnofsky performance scale (KPS) score was evaluated at each consultation. Acute toxicity was assessed according to the Common Terminology Criteria for Adverse Events (CTCAE) v5.0.

**Fig. 1:**
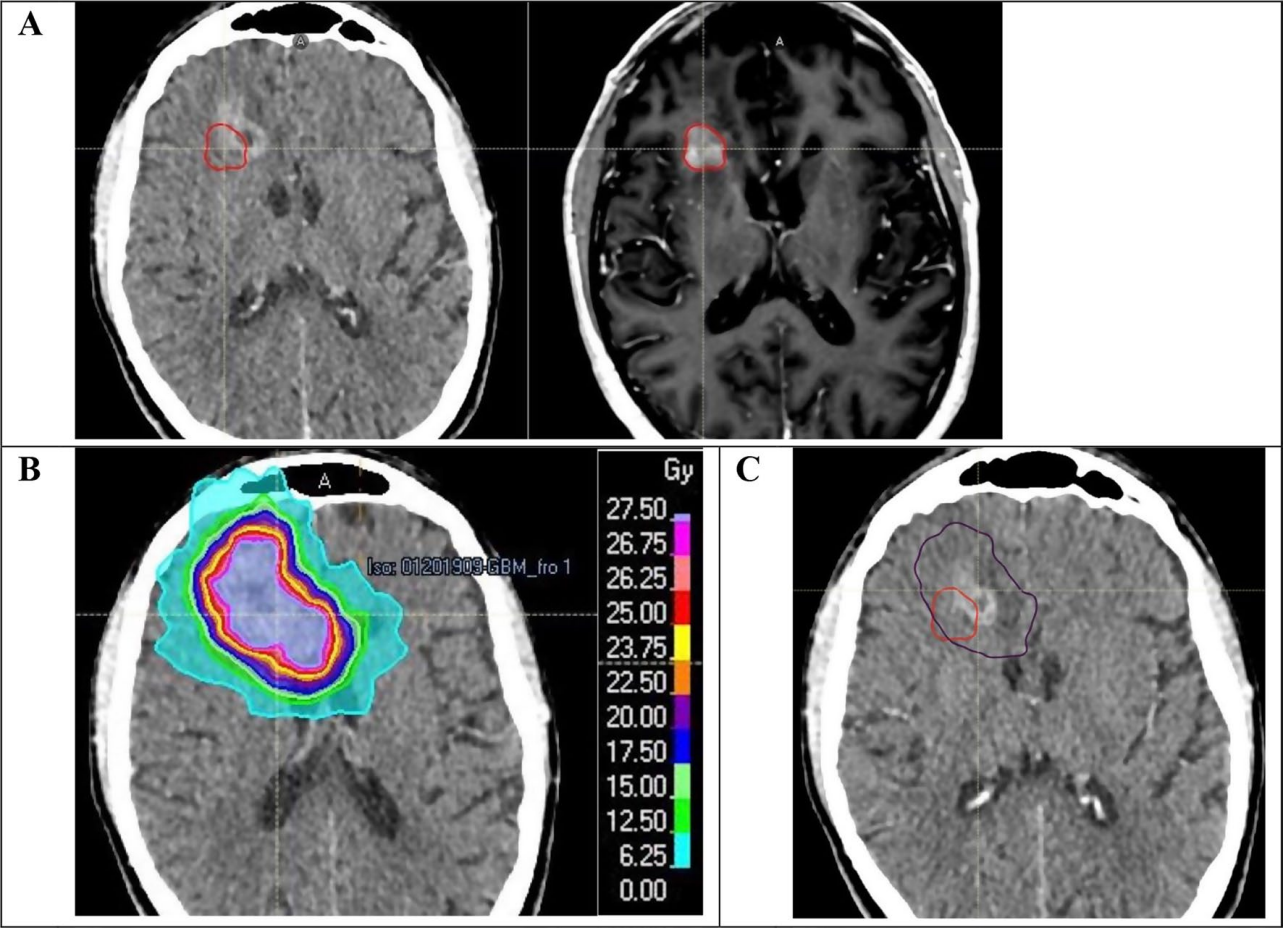
**A**. MRI showing post-fSRT (delineated in red) recurrence on the right, matched with the fSRT treatment planning scan on the left; **B**. fSRT treatment plan; **C**. recurrence post-fSRT (red) and volume which received at least 20 Gy (purple) on the fSRT treatment planning scan

To determine the recurrence location, the MRI that allowed to conclude to a radiographic progression for each patient was imported and fused to the fSRT treatment plan, in the software used to delineate the volumes, RayStation© (v11B, RaySearch Laboratories AB). The gross tumor volume of the recurrence (GTV-R) was delineated and included all the T1-enhanced lesions, based on the MRI. This volume was validated by an expert neuroradiologist, blinded to the previous reirradiation isodose volume. A second volume called “V20Gy” was created, corresponding to the volume which received at least 20 Gy (Fig. 1), namely 80% of the prescribed dose, to be consistent with other studies [17, 18, 21, 22]. A Boolean operation was performed to determine the common volume between GTV-R and V20Gy. The recurrences were then classified into three categories:

**Fig. 2:**
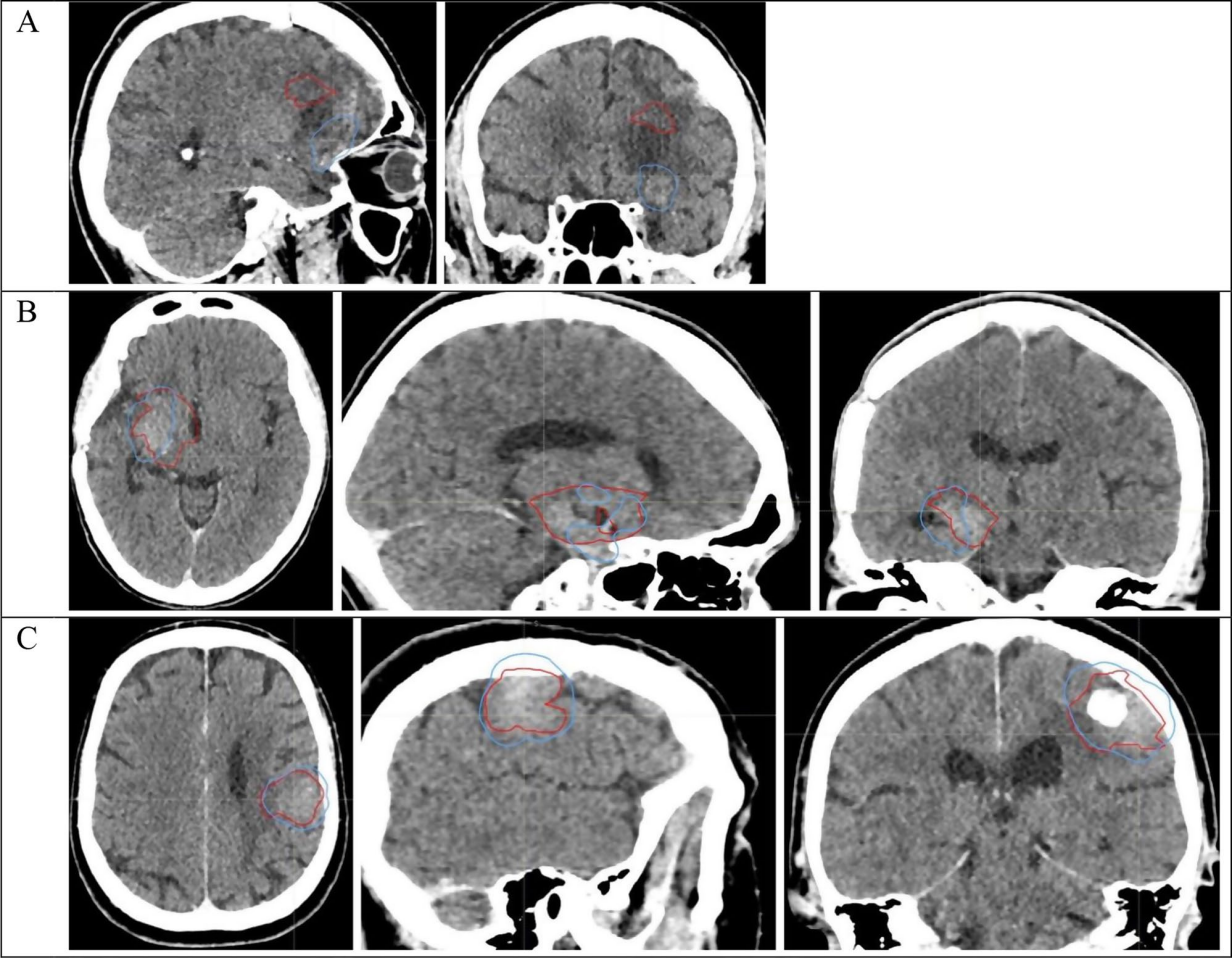
Recurrence post-fSRT (GTV-R, in red) and volume which received at least 20 Gy (V20Gy, in blue) on the fSRT treatment planning scan in axial, coronal and sagittal cross-sections. **(A)** “out-field” recurrence with a common volume of 0% between GTV-R and V20Gy; **(B)** “marginal” recurrence with a common volume of 72% between GTV-R and V20Gy; **(C)** “in-field” recurrence with a common volume of 89%

- In-field if more than 80% of the GTV-R volume was contained into the V20Gy volume, i.e., if more than 80% of the tumor recurrence volume was contained in the 20 Gy isodose surface.
- Marginal if 20–80% of the GTV-R volume was contained into the V20Gy volume.
- Out-field if less than 20% of the GTV-R volume was contained into the V20Gy volume.

For ease of understanding in this study, the recurrence that was reirradiated by Cyberknife© will be called “fSRT recurrence”, and the recurrence post-fSRT (the locations of interest in this study) will be simply called “recurrence”.

### Study endpoints

The primary study endpoint was the determination of a pattern of recurrence after reirradiation by fSRT. The secondary study endpoints were progression-free survival (PFS) after fSRT reirradiation, overall survival from first glioblastoma diagnosis (OS) and from reirradiation (OS-R).

### Statistical analysis

Qualitative parameters were described by frequency and percentage, and quantitative parameters by median and range.

Survival analysis and tumor control were described using the non-parametric Kaplan-Meier method from the first day of treatment by fSRT. OS was determined from the day of initial surgery. We used the Log-rank test to compare survival data. To correct for multiple tests, p-values were adjusted using the Benjamini-Hochberg (BH) method.

Risk factors were evaluated by univariate and multivariate Cox models. Parameters with p-value less than 0.1 were included in a multivariate Cox model (stepwise selection). Results were presented as adjusted hazard ratios (HR) with 95% confidence intervals.

Analyses were performed with RStudio Software, version 2022.07.2 + 576. Statistical significance was set at 0.05.

## Results

62 patients matched the inclusion criteria. Patients’ characteristics are described in Table 1. At the first diagnosis of glioblastoma, all patients underwent the same and complete chemoradiation treatment after surgery or biopsy. The fSRT recurrence was the first recurrence in 53 patients (85.5%). The median time between the end of radiochemotherapy and first day of fSRT was 10.6 months (range 2.4–58.1 months). Reirradiation treatment characteristics are described in Table 2. At recurrence after fSRT, lesions were multifocal in 21 patients (33.8%).

**Table 1:** Patient characteristics at diagnosis, recurrence (treated by fSRT), 3 months after fSRT, and recurrence after fSRT

**At diagnosis**	
Age (years)	59 (38–78)
Female	28 (45.2)
Surgical procedure	
- Gross total resection	15 (24.2)
- Subtotal resection	29 (46.8)
- Biopsy	18 (29)
Adjuvant systemic therapy	
- Temozolomide	50 (80.6)
- Bevacizumab	1 (1.6)
- Nivolumab	1 (1.6)
- Temozolomide + Bevacizumab	7 (11.3)
- Temozolomide + Nivolumab	1 (1.6)
- Temozolomide ± Pazopanib	1 (1.6)
- None	1 (1.6)
Subventricular zone invasion	
- Yes	10 (16.1)
- No	52 (83.9)
**At fSRT recurrence**	
Age (years)	60 (40–78)
KPS (%)	
- ≥ 70	59 (95.2)
- < 70	3 (4.8)
Number of previous recurrences/treatments	
- 0	53 (85.5)
- 1	8 (12.9)
- Fotemustine + Bevacizumab	2 (3.2)
- Lomustine + Bevacizumab	1 (1.6)
- Surgery + radiochemotherapy with Temozolomide	1 (1.6)
- Temozolomide	3 (4.8)
- Surgery	1 (1.6)
- 2	1 (1.6)
- Temozolomide then Fotemustine + Bevacizumab	1 (1.6)
Systemic therapy	
- Yes	27 (43.5)
- Fotemustine + Bevacizumab	4 (6.5)
- Temozolomide + Bevacizumab	2 (3.2)
- Belustine + Bevacizumab	1 (1.6)
- Bevacizumab	4 (6.5)
- Temozolomide	14 (22.6)
- Muphoran	1 (1.6)
- Lomustine	1 (1.6)
- No	35 (56.5)
Multifocality	
- Yes	12 (19.4)
- No	50 (80.6)
Steroids	
- Yes	18 (29)
- No	44 (71)
Toxicity	
- None	46 (74.2)
- Grade 1	12 (19.4)
- Grade 2	3 (4.8)
- Grade 3	1 (1.6)
**At 3 months after fSRT**	
KPS (%)	
- ≥ 70	50 (80.6)
- < 70	12 (19.4)
Steroids	
- Yes	35 (56.5)
- No	26 (41.9)
**At recurrence (after fSRT)**	
KPS (%)	
- ≥ 70	43 (69.4)
- < 70	19 (30.6)

Result: median (minimum-maximum) or frequency (percentage)

fSRT, fractionated stereotactic reirradiation; KPS, Karnofsky Performance Status

**Table 2:** Reirradiation treatment characteristics

Total dose (Gy)	25 (25–25)
Prescription isodose (%)	80 (80–80)
Numbers of beams	95 (38–164)
GTV (cc)	5.29 (0.28–41.75)
PTV (cc)	10.55 (0.98–50.69)
GTV-PTV margin (mm)	2 (1–3)

Result: median (minimum-maximum)

GTV, gross tumor volume; PTV, planning target volume; Cc, cubic centimeter; mm, millimeter

The location of recurrences after fSRT was out-field in 43.5% (27/62 patients, Fig. 2A), marginal in 40.3% (25/62 patients, Fig. 2B), and in-field in 16.1% (10/62 patients, Fig. 2C). In the out-field group, there were 15 patients (55.6%) with no common volume at all between the recurrence volume and the reirradiated volume.

We tested different predictive factors concerning the location of recurrences (Table 3). There were no significant predictive factors for the in-field recurrences. Moreover, we found that the size of the GTV-PTV margin (2–3 mm compared with 1 mm, HR = 0.38 [0.15–0.95], *p* = 0.037) and a PTV volume of ≥ 36 cc (HR = 5.18 [1.06–25.3], *p* = 0.042) were significantly associated with marginal recurrences in both univariate and multivariate analysis. Finally, we found that an age at initial treatment of ≥ 60 years (HR = 3.06 [1.17–8.01], *p* = 0.023) and having one or more previous recurrences (HR = 5.29 [1.70–16.5], *p* = 0.004) were significantly associated in multivariate analysis with out-field recurrences.

In the subgroup of patients who had recurrences at least 90 days after reirradiation, the localization of recurrences was “out-field” in 35.1% (13/37 patients), “marginal” in 56.8% (21/37 patients), and “in-field” in 8.1% (3/37 patients).

**Table 3:** Predictive factors in-field, marginal or out-field recurrence after fSRT: univariate and multivariate analysis

	In-field				Marginal					Out-field			
Statistical analysis	Univariate		Multivariate		Univariate		Multivariate		Univariate			Multivariate	
Age at initial treatment ≥ 60	HR = 0.45 [0.12–1.73]	*p* = 0.2			HR = 0.74 [0.34–1.64]	*p* = 0.5			HR = 1.83 [0.84–3.98]		*p* = 0.13	**HR = 3.06 [1.17–8.01]**,	*p* = 0.023
Type of initial surgical procedure													
- Biopsy	-	-			-	-			-		-		
- Subtotal resection	HR = 0.72 [0.17–3.01]	*p* = 0.7			HR = 1.54 [0.53–4.44]	*p* = 0.4			HR = 0.58 [0.17–1.98]		*p* = 0.4		
- Gross total resection	HR = 0.24 [0.05–1.22]	*p* = 0.085			HR = 1.23 [0.46–3.34]	*p* = 0.7			HR = 1.50 [0.62–3.64]		*p* = 0.4		
Invasion of SVZ at diagnosis	HR = 0.58 [0.07–4.57]	*p* = 0.6			HR = 0.79 [0.24–2.63]	*p* = 0.7			HR = 1.95 [0.78–4.83]		*p* = 0.2		
Multifocality of recurrence treated by fSRT	HR = 3.56 [1.00–12.7]	*p* = 0.050	HR = 3.25 [0.89–11.9]	*p* = 0.075	HR = 0.93 [0.32–2.70]	*p* = 0.9			HR = 0.70 [0.24–2.03]		*p* = 0.5		
Adjuvant systemic therapy at recurrence treated by fSRT	HR = 0.54 [0.14–2.09]	*p* = 0.4			HR = 1.38 [0.63–3.03]	*p* = 0.4			HR = 1.23 [0.58–2.64]		*p* = 0.6		
KPS score at recurrence treated by fSRT ≥ 70	HR = 0.29 [0.04–2.29]	*p* = 0.2			HR = 25 655 552 [0 – inf]	*p* > 0.9	HR = 1.05 [1.00–1.10]	*p* = 0.051	HR = 0.26 [0.06–1.16]		*p* = 0.078	HR = 0.97 [0.93–1.00]	*p* = 0.084
Existence of one or more previous recurrences	HR = 0.70 [0.09–5.49]	*p* = 0.7			HR = 0.62 [0.15–2.63]	*p* = 0.5			**HR = 2.70 [1.08–6.72]**		*p* = 0.033	**HR = 5.29 [1.70–16.5]**	*p* = 0.004
GTV volume	HR = 1.04 [0.99–1.10]	*p* = 0.13			HR = 1.03 [0.98–1.09]	*p* = 0.2			HR = 0.97 [0.91–1.003]		*p* = 0.3		
GTV volume ≥ 17 cc	HR = 2.22 [0.47–10.5]	*p* = 0.3			HR = 2.50 [0.73–8.52]	*p* = 0.14			HR = 0.68 [0.16–2.89]		*p* = 0.6		
PTV volume	HR = 1.04 [1.00–1.09]	*p* = 0.055	HR = 1.04 [0.99–1.08]	*p* = 0.089	HR = 1.02 [0.98–1.06]	*p* = 0.2			HR = 0.97 [0.93–1.02]		*p* = 0.2		
PTV volume ≥ 36 cc	HR = 2.43 [0.31–19.3]	*p* = 0.4			**HR = 6.23 [1.35–28.7]**	*p* = 0.019	**HR = 5.18 [1.06–25.3]**	*p* = 0.042	HR = 0.00 [0.00 – inf]		*p* > 0.9		
GTV-PTV margin 2 and 3 mm vs. 1 mm	HR = 256 164 988 [0 – inf]	*p* > 0.9			**HR = 0.42 [0.18–0.98]**	*p* = 0.044	**HR = 0.38 [0.15–0.95]**	*p* = 0.037	HR = 1.85 [0.56–6.18]		*p* = 0.3		

SVZ, SubVentricular Zone; fSRT, fractionated Stereotactic RadioTherapy; KPS, Karnofsky Performance Status; HR, Hazard Ratio; GTV, Gross Tumor Volume; PTV, Planning Target Volume; cc, cubic centimeter

HR computed for an increase of 10 cc

The median PFS from fSRT was 3.4 months (95% CI 2.9–4.8 months). We found that an age at initial treatment of ≥ 65 years (HR = 0.56 [95% CI 0.32–0.98], *p* = 0.041), a KPS score at recurrence ≥ 70% (HR = 0.27 [95% CI 0.08–0.89], *p* = 0.032), and having one or more previous recurrences (HR = 2.44 [95% CI 1.16–5.13], *p* = 0.019), were significantly associated with PFS in univariate analysis. A PTV volume of ≥ 35 cc (HR = 2.78 [95% CI 0.97–7.97], *p* = 0.056) was close to being significant. We explored other predictive factors for PFS, which were not significant, including the type of initial surgical procedure, invasion of SVZ (subventricular zone) at diagnosis, multifocality of the recurrence, use of corticosteroids at recurrence, adjuvant systemic therapy at recurrence, GTV volume, GTV-PTV margin, and time between the end of radiochemotherapy and first day of fSRT. In multivariate analysis, we found that a KPS score of ≥ 70% at recurrence was significantly associated with a better PFS (HR = 0.27 [95% CI 0.08–0.93], *p* = 0.038), whereas a PTV volume ≥ 35 cc (HR = 3.61 [95% CI 1.23–10.6], *p* = 0.02) and having one or more previous recurrences (HR = 2.32 [95% CI 1.07–5.05], *p* = 0.033) were significantly associated with a decrease of the PFS. Age at initial treatment (HR = 0.64 [95% CI 0.36–1.16], *p* = 0.14) was not significant in multivariate analysis.

The median OS from first glioblastoma diagnosis was 25.7 months (95% CI 22.2–32 months), and from fSRT (OS-R) was 10.8 months (95% CI 8.97–14.8 months). The median OS without institutionalization from fSRT was 9.24 months (95% CI 8.1–14.1 months).

## Discussion

In this large cohort of patients, we observed mostly out-field recurrences of glioblastoma after reirradiation. This is a completely different result from the other studies that studied recurrence following reirradiation, whereby they all found a majority of in-field recurrences [17, 18, 21, 22]. Several reasons can explain this difference. First, the definitions of the location of recurrences were different for two of these studies: in-field recurrences were defined by the volume of recurrence inside the 50% isodose prescription line [21, 22]. The definition we used was first described in studies concerning the pattern of recurrence of glioblastomas after initial treatment [23, 24], and then used in more recent studies about the pattern of relapse after reirradiation [17, 18]. Second, the modality of reirradiation was not the same as in our study, with fSRT delivered by CyberKnife^®^ (25 Gy in 5 fractions, isodose of prescription 80%): one used SRS, which is a unique fraction of radiotherapy [22], the second one used conventional radiation therapy [17], and the third one used fSRT not delivered by a dedicated stereotactic device [18]. In fact, for our patients, the reirradiated area received at least 100 Gy in terms of EDQ2Gy: 60 Gy at the initial treatment, and at least 40 Gy at reirradiation (EQD2Gy of 25 Gy in 5 fractions), with a higher dose in the center of the volume due to the prescription to the normalized 80% isodose. It is known that there is increased radionecrosis risk in such areas [25], which may explain the very few in-field recurrences in our cohort, given the high frequency of asymptomatic radionecrosis we have observed. The modality of reirradiation may also explain our large proportion of marginal recurrences, by the absence of delineation of a clinical target volume (CTV), leading to a smaller margin between GTV and PTV (i.e. between the tumor and the volume effectively treated); there is also the lack of consideration of T2/FLAIR non-enhanced lesions in the treated volume. The recommendations for fSRT treatment only concern small lesions, which are more often T1-enhanced lesions (high-grade lesions), while evolutive T2/FLAIR lesions tend to lead to the resumption of a systemic treatment [26].

We found that a GTV-PTV margin of 1 mm was a significant predictive factor for marginal recurrences (versus a 2–3 mm margin), which is consistent with the fact that a 1 mm margin is not appropriate for a CyberKnife© treatment, considering the positioning uncertainties. This 1 mm margin is used for Gamma Knife treatments with a stereotactic frame, which reduces these uncertainties. We also found that a PTV volume of ≥ 36 cc was significantly associated with marginal recurrences. This is consistent with the literature: Nyazi et al. found a poorer local control for larger lesions (PTV > 75 cc) [17].

Concerning out-field recurrences, we found an association with older age at diagnosis and existence of one or more previous recurrences, which are also poor prognosis factors, thus reflecting a more advanced disease. Other predictive factors were found in other studies, such as the association of a high-dose of irradiation and the use of bevacizumab with out-field recurrences [21]. We were unable to study this type of predictive factor because of systemic agent heterogeneity, in mono- or multitherapy.

Concerning the risk of recurrences being pseudoprogression instead, we supposed that it would induce more in-field recurrences. We found 8.1% of in-field recurrences in patients who had their recurrence at least 90 days after fSRT reirradiation, which is close to proportion of in-field recurrences in the complete cohort. Given the similar proportions in the two groups, we assumed the possibility of a bias to increase our statistical power, with the higher proportions of marginal and out-field recurrences in the two populations (compared to in-field).

We compared our survival data with that in the literature, specifically concerning recurrent glioblastoma treated by fSRT with a dedicated stereotactic device (Table 4). A strict comparison was difficult because of the heterogeneity of inclusion criteria in these studies, with inclusion of grade III gliomas for some, and different designs regarding dose and fractionation regimens, normalized isodose prescriptions, and especially systemic treatment at recurrence. In most of these studies, patients received a systemic treatment at recurrence, whereas more than half of our patients did not receive any systemic agent. For these reasons, the PFS of our cohort is lower than those reported in the literature and, more specifically, in those concerning grade IV gliomas, which could suggest our treatment strategy was less effective, the main difference being the delayed administration of a systemic agent. However, the OS and the OS-R are similar to those reported, so there is no clear case for a particular strategy being better.

**Table 4:** Review of studies about glioblastoma recurrence treated by fSRT with dedicated stereotactic devices

Author/year of publication	Prospective/ Retrospective	Number of patients	Tumor grade	Dose/fractionation (median) Isodose of prescription	Systemic treatment at recurrence	Free interval between RT	OS	OS-*R*	PFS	PTV (cc)
Wurm et al. 2006 [27]	P	25 (20 grade IV)	IV, III	25 Gy/5fr Isodose 80%	Topotecan	12 m (IV)	27 m (IV)	7.9 m (IV)	5.6 m (IV)	NR
Villavicencio et al. 2009 [28]	R	26	IV	26 Gy/2fr Isodose 78%	Yes (no precision)	NR	21 m	7 m	NR	7 (0.4–48.5)
McKenzie et al. 2013 [29]	P	33	IV, III	30 Gy/5fr Isodose 100%	TMZ, Bev, Irinotecan, others	NR	22 m	8.6 m	62% à 6 m	8.54 (0.4-46.56)
Minniti et al. 2013 [30]	R	54 (38 grade IV)	IV, III	30 Gy/5fr Isodose 90%	TMZ	15.5 m	27.9 m	11.4 m (IV)	4 m (IV)	30.3 (12.3–53.4)
Ertas et al. 2014 [31]	R	42	IV, III	18 Gy/3fr	NR	NR	28 m (IV)	8 m (IV)	NR	42 (3.6–144)
Hasan et al. 2015 [32]	P	19	IV	25 Gy/5fr Isodose 73%	Bev, TMZ, antiEGFR	NR	NR	5.3 m	NR	35 (0.9-151.7)
Antoni et al. 2016 [21]	R	20	IV, III, gliosarcoma	31.2 Gy/6fr Isodose 80%	TMZ, Bev, Bev + Irinotecan	18.3 m	NR	17.7 m	12 m	2.71 (0.24–28.4)
Jia et al. 2016 [33]	R	21	IV	33 Gy/3fr Isodose 80%	Bev, TMZ, TMZ + Bev, Erlotinib	9.6 m	NR	9.3 m	5.2 m	NR
Gigliotti et al. 2018 [34]	R	25 (20 grade IV)	IV, III	25 Gy/5fr Isodose 80%	TMZ/veliparib, Bev/TMZ, Bev	18 m	39 m	9 m	57% at 6 m, 39% at 12 m	9.83 (0.55–92.78)
Guan et al. 2021 [35]	R	70 (49 grade IV)	IV, III	24 Gy/4fr Isodose 70%	TMZ, Bev, TMZ + Bev	NR	NR	14.6 m (IV)	6.8 m (IV)	16.68 (0.81-121.96)
Yazici et al. 2014 [36]	P	37	IV	30 Gy/5fr	TMZ, Beva, CCNU	15 m	35.5 m	10.6 m	7.9 m	NR
Greenspoon et al. 2014 [37]	P	31	IV	25–35 Gy/5fr	TMZ	NR	NR	9 m	7 m	NR
Pinzi et al. 2015 [38]	R	128 (88 grade IV)	IV, III	19 Gy/2fr	NR	15 m	25 m	10	NR	11 (0.63–121)
Margulies et al. 2023	R	62	IV	25 Gy/5fr Isodose 80%	TMZ, Bev, TMZ + Bev, Fotemustine + Bev, CCNU	10.6 m	25.7 m	10.8 m	3.4 m	10.55 (0.98–50.69)

RT, radiation therapy; NR, not referenced; TMZ, temozolomide; Bev, bevacizumab; m, months; cc, cubic centimeter

Concerning predictive factors, we found that a KPS of ≥ 70% and a PTV of < 35 cc were significantly associated with a better PFS in multivariate analysis, which is consistent with the literature [26, 39].

The strength of our study comes from our large cohort of patients, all of whom had grade IV gliomas at diagnosis, who all received the same radiation dose and fractionation regimen, at initial treatment and at recurrence. This is also, to our knowledge, the first dosimetric study concerning this specific modality of reirradiation of glioblastoma.

However, it has some limitations, including its retrospective design. We also did not consider the multifocality of recurrences after fSRT, adding all the volumes in the calculation of the common volume between GTV-R and V20Gy, which may have caused a bias in the classification of the location of the recurrence. The lack of a consensual definition of local control after reirradiation by fSRT is also a weakness, raising the question about marginal recurrences: are they really from the border of the reirradiated area, or are they in-field recurrences with a substantial increase, outgrowing the initial area and reaching the periphery? Finally, we included the use of adjuvant systemic treatment of reirradiation in our analysis and not specifically Bevacizumab, because of the limited number of concerned patients and the lack of statistical power, which can also cause a bias in the interpretation of our results.

Our study highlights the absence of delineation of a clinical target volume for the CyberKnife© treatment, which may decrease marginal recurrences of glioblastoma and warrants further study. There may be also be potential contributions from single-photon emission computed tomography (SPECT), which may help to target al.l the high grade recurrent lesions [40].

## Conclusion

Despite the poor prognosis of glioblastoma, our study showed that reirradiation of glioblastoma by fSRT with 25 Gy in 5 fractions (to the 80% isodose) provides good local control, with recurrences occurring mostly outside of the reirradiated area.

## Data Availability

No datasets were generated or analysed during the current study.
